# Iron Status in Newly Diagnosed *β*-Thalassemia Major: High Rate of Iron Status due to Erythropoiesis Drive

**DOI:** 10.1155/2021/5560319

**Published:** 2021-04-16

**Authors:** Susi Susanah, Lulu Eva Rakhmilla, Mohammad Ghozali, Jessica Oktavianus Trisaputra, Octawyana Moestopo, Yunia Sribudiani, Ponpon S. Idjradinata, Ani Melani Maskoen

**Affiliations:** ^1^Department of Child Health, Hematology-Oncology Division, Dr. Hasan Sadikin General Hospital/Faculty of Medicine, Universitas Padjadjaran, Bandung 40161, Indonesia; ^2^Department of Public Health, Epidemiology and Biostatistics Division, Faculty of Medicine, Universitas Padjadjaran, Bandung 40161, Indonesia; ^3^Department of Biochemistry and Molecular Biology, Working Group of Clinical Genetics, Faculty of Medicine, Universitas Padjadjaran, Bandung 40161, Indonesia; ^4^Faculty of Medicine, Universitas Padjadjaran, Bandung 40161, Indonesia

## Abstract

**Background:**

Iron overload in severe *β*-thalassemia is a serious complication that occurs during the course of the disease. Information about the iron status during initial illness with *β*-thalassemia major seemed to be limited. This study is aimed at analyzing iron status, serum hepcidin, and growth differentiation factor 15 (GDF15) levels in newly diagnosed *β*-thalassemia major.

**Methods:**

A case-control study was performed at Dr. Hasan Sadikin General Hospital, which included 41 children with newly diagnosed *β*-thalassemia major. Age- and sex-matched controls were enrolled. The subjects had no blood transfusion, had normal liver function, and had no sign of inflammation. The groups were compared in terms of the levels of hemoglobin (Hb), serum ferritin (SF), transferrin saturation (TS), serum hepcidin, and GDF15 as iron homeostasis parameters.

**Results:**

Of the 41 newly diagnosed *β*-thalassemia major patients, those who were less than 24 months old had significantly lower median Hb levels than controls (5.0 vs. 11.7 g/dL, *P* < 0.001). The median SF and TS levels were significantly higher than those in controls (315.0 vs. 29.0 ng/mL, *P* < 0.001; 70.6 vs. 16.5%, *P* < 0.001), and median hepcidin was at the normal limit, but the value was higher in patients (251.0 vs. 123.1 ng/mL, *P* < 0.001). The median GDF15 level was significantly higher in patients (2,095.3 vs. 342.4 pg/mL, *P* < 0.001). There was a positive correlation between SF-TS, SF-hepcidin, TS-hepcidin, SF-GDF15, TS-GDF15, and hepcidin-GDF15 (*P* < 0.001).

**Conclusion:**

In newly diagnosed *β*-thalassemia major, an increase in iron status occurred. This may be caused by increased iron absorption due to massive erythropoietic activity, characterized by an increase in GDF15 levels, which does not cause hepcidin suppression. The iron homeostasis response seems to be physiologically indicated by a tendency to increase hepcidin levels.

## 1. Introduction

Beta-thalassemia is hereditary anemia that results from defects in globin genes that are inherited in an autosomal recessive pattern. Severe forms could be *β*-thalassemia major which is predicted to require regular blood transfusions or to be transfusion-dependent thalassemia (TDT) cases [1, 2]. The remarkable accumulation of excess *α*-globin chains in the erythroids of *β*-thalassemia major leads to ineffective erythropoiesis (IE), which is defined by an erythroid defect with the reduction in mature erythrocytes, although erythroid precursors greatly increase in number [3–5]. In *β*-thalassemia, IE is characterized by expansion, limited differentiation, and premature death of erythroid precursors [4]. Reduced RBCs could lead to anemia and hypoxia that stimulate erythropoietin (EPO) production to induce erythropoiesis. Over time, the combination of hypoxia, increased EPO, and IE creates a vicious circle that induces massive erythroblast expansion in the bone marrow. The remarkable accumulation of excess *α*-globin chains in the erythroids due to *β*-thalassemia major results in IE inducing the release of certain cytokines, such as growth differentiation factor 15 (GDF15), which might suppress hepcidin [1, 6–10]. GDF15 is a protein produced by erythroid precursor erythroblasts, which can be measured. GDF15 mediates messenger ribonucleic acid (mRNA) hepcidin suppression in anemia due to IE in vitro [11]. Low hepcidin will lead to increased iron absorption in the gastrointestinal tract, and chronic transfusions are responsible for the state of iron overload in *β*-thalassemia major [6].

Iron overload is the principal and multifaceted complication of *β*-thalassemia major [1, 6]. Disruption of iron homeostasis leads to the deposition of iron in vital organs and becomes the main cause of death in patients with *β*-thalassemia major [12]. Serum ferritin (SF) and transferrin saturation (TS) levels can indicate the iron status in the human body, although the liver iron concentration (LIC) is considered to be the most representative parameter for the iron status in the body [2, 13, 14]. Practically, SF and TS levels were used to determine the iron status in thalassemia [2].

Iron status is orchestrated by a regulatory system known as homeostasis, which involves the interaction of many factors [8, 15, 16]. Iron homeostasis is physiologically regulated by controlling its absorption, storage, and recycling. Several factors that induce iron absorption are iron storage, the erythropoietic activity level in the bone marrow, the hemoglobin (Hb) level, the oxygen concentration in blood, and inflammatory cytokines. Hepcidin, the peptide hormone that is mostly synthesized in hepatocytes, is the master regulator of iron homeostasis that controls serum iron through the degradation of ferroportin in enterocytes and macrophages [17]. The binding of ferroportin to hepcidin promotes its phosphorylation, internalization, and degradation, which limits the entry of iron into circulating enterocytes or macrophages [14, 16]. Under low hepcidin expression, iron uptake from macrophages and intestinal iron absorption are increased, while high hepcidin expression inhibits iron uptake and absorption [6, 16, 18]. The regulation of hepcidin is induced by iron deficiency, expansion of erythropoiesis, anemia, hypoxia, and possibly other factors [17, 19]. Massive IE in *β*-thalassemia major conditions will increase iron demand in the body, which sends a signal to increase absorption. This condition leads to an iron overload state in *β*-thalassemia major [11, 20, 21].

Several previous studies have reported serum hepcidin levels, iron status, and GDF15 levels in multitransfused *β*-thalassemia patients. However, information on these protein expression levels and iron status in newly diagnosed patients with *β*-thalassemia major is very limited [9, 22–25]. This study was conducted to analyze the iron status, serum hepcidin, and GDF15 levels in newly diagnosed patients with *β*-thalassemia major and the correlation between variables.

## 2. Materials and Methods

A case-control study was performed at the Department of Child Health, Dr. Hasan Sadikin General Hospital, a top referral hospital for West Java Province in Indonesia. The cases were recruited consecutively, while the controls were selected with the premise of considering healthy children who met the requirements of the research.

### 2.1. Ethics and Consent

Approval from the Institutional Ethics Committee at Dr. Hasan Sadikin General Hospital was obtained, and the study was conducted according to the principles of the Declaration of Helsinki. Participation of the subjects was requested from patients' parents/guardians by written informed consent.

### 2.2. Subjects

A case was defined as a patient of any age and sex with newly diagnosed *β*-thalassemia major and was recruited based on conventional clinical and hematologic criteria who had not received any blood transfusion. Controls who were individually matched by age and sex were from the community without a history of thalassemia. Controls were drawn from primary healthcare in Bandung consecutively from their arrival at the facility. Demographic information (age and sex) were obtained from the immediate guardians, which were usually the mothers. Fifty subjects with newly diagnosed *β*-thalassemia major were recruited in this study and matched by age and sex 1 : 1 with the controls. Both groups underwent a full laboratory examination with no sign of inflammation with normal C-reactive protein (CRP) and interleukin-6 (IL-6) and normal liver function based on aspartate transaminase (AST) and alanine transaminase (ALT). Subjects with improper laboratory samples, so that the results obtained from the sample were unsuitable, were excluded. Assessment of nutritional status was carried out by measuring body weight and height according to the age and then plotted into the WHO child growth standard. Nutritional status was classified based on WHO BMI-for-age as severe malnutrition (BMI-for-age < −3*z*-score), mild malnutrition (BMI-for-age ≥ −3 and <-2 *z*-score), and normal nutritional status (≥-2 and ≤+1 *z*-score).

### 2.3. Laboratory Examinations

A venous blood sample was collected prior to the first transfusion of packed red cells. Hematologic analysis was performed using an automated blood cell analyzer (Sysmex XN 1000 A), and Hb analysis was confirmed by high-performance liquid chromatography (HPLC) (Bio-Rad, USA) at the Department of Clinical Pathology, Dr. Hasan Sadikin General Hospital. Chemical immunoassay (Reagent, Siemens EXL 200 Dimension, USA) was used to measure liver function (AST and ALT), while particle-enhanced turbidimetric immunoassay (Dimension, USA) for CRP and ELISA (Elabscience, USA) for IL-6 levels. Iron status was measured by using enzyme immunoassays (Centaur XPT, Siemens, USA), while serum hepcidin levels were measured by human hepcidin using competitive enzyme-linked immunosorbent assay (C-ELISA) (R&D Systems, Minneapolis, MN, USA). The measurement of GDF15 levels was performed by using the quantitative sandwich enzyme immunoassay technique method (ELISA–Quantikine, human GDF15 immunoassay from R&D Systems, Inc., USA). The normal range for GDF15 is 289–1,096 pg/mL, and hepcidin is 12.5–400 ng/mL. The normal range for CRP is 0–6 mg/L, IL-6 is 0–16.4 pg/mL, ALT is 5–45 U/L, and AST is 20–63 U/L.

### 2.4. Statistical Analysis

The data were analyzed using SPSS software version 25.0 (IBM Corp., Armonk, NY). Descriptive results for categorical variables are described as numbers and percentages, while continuous variables are described as the mean ± SD or median (range). Age, sex, nutritional status, Hb, SF, TS, hepcidin, GDF15, ALT, AST, IL-6, and CRP between both groups were compared using independent Student's *t*-tests and Mann-Whitney tests. Spearman's correlation was used to define all correlations between SF-TS, SF-hepcidin, SF-GDF15, hepcidin-GDF15, hepcidin-TS, and hepcidin-SF. A *P* value < 0.05 was considered statistically significant in all analyses.

## 3. Results

Forty-one subjects with newly diagnosed *β*-thalassemia major and 50 healthy controls were included in this study. Nine subjects with newly diagnosed *β*-thalassemia major were excluded because of improper laboratory samples, for which sample results were unsuitable. All the subjects originated from the Sundanese ethnic group, with normal liver function and no signs of infection or inflammation, and were characterized by normal CRP and IL-6 serum levels.

All of the subjects were less than 24 months old, 80% were under 1 year old, and 73% were well nourished. Ninety-five percent of cases had severe anemia with Hb levels < 7 g/dL. The median SF and TS levels were significantly higher in the cases than in the controls (315.0, range 83.6-1,071.0 ng/mL and 70.6, range 28.5-98.7%, respectively, *P* < 0.001). The median serum hepcidin level in patients and controls was somehow within the normal limit, but the value was higher in patients (251.0, range 114.5-363.5 ng/mL) than in controls (123.1, range 5.6-349.5 ng/mL; *P* < 0.001). All of the patients showed higher erythropoietic activity based on the level of GDF15, which was significantly higher in the case group than in the control group (median 2,095.3 vs. 342.4, range 149.3-9,586.3 vs. 350.08-436.99 pg/mL; *P* < 0.001), as shown in Table 1. Based on the inclusion criteria, both the case and control groups had normal CRP (2.9 ± 1.7 mg/L, range 0.2–7.7 vs. 1.6 ± 1.6 mg/L, range 0.2–6.0), normal IL-6 (8.7 ± 9.1 pg/mL, range 0.0–45.7 vs. 2.3 ± 2.5 pg/mL, range 0.0–7.1), normal AST (42.2 ± 13.7 U/L, range 21.0–95.0 vs. 40.5 ± 6.8 U/L, range 26.0–56.0), and normal ALT (23.9 ± 10.2 U/L, range 5.0–60.0 vs. 33.8 ± 8.0 U/L, range 17.0–45.0).

**Figure 1:**
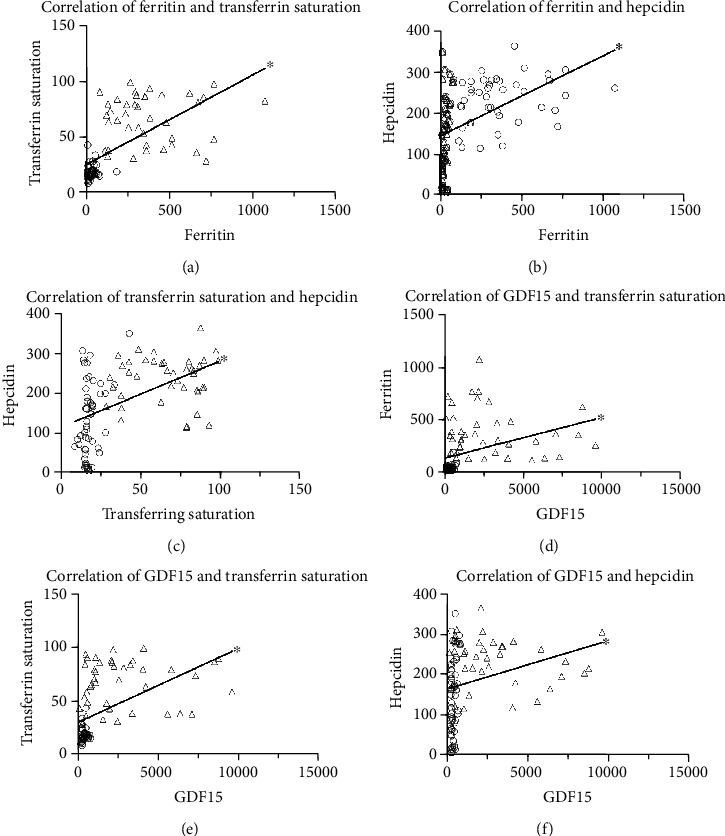
Positive correlation of (a) SF and TS, *P* < 0.001, *r* = 0.794, and *Y* = 0.08029∗*X* + 24.99; (b) SF and hepcidin, *P* < 0.001, *r* = 0.594, and *Y* = 0.1958∗*X* + 144.1; (c) TS and hepcidin, *P* < 0.001, *r* = 0.526, and *Y* = 1.662∗*X* + 114.3; (d) SF and GDF15, *P* < 0.001, *r* = 0.616, and *Y* = 0.03888∗*X* + 130.7; (e) TS and GDF15, *P* < 0.001, *r* = 0.658, and *Y* = 0.08029∗*X* + 24.99; and (f) GDF15 and hepcidin, *P* < 0.001, *r* = 0.453, and *Y* = 0.01204∗*X* + 163.1. Control: circle. Case: triangle. SF = serum ferritin; TS = transferrin saturation; GDF15 = growth differentiation factor 15.

There was a positive correlation between SF and TS (*P* < 0.001, *r* = 0.794), SF and hepcidin (*P* < 0.001, *r* = 0.594), TS and hepcidin (*P* < 0.001, *r* = 0.526), SF and GDF15 (*P* < 0.001, *r* = 0.616), TS and GDF15 (*P* < 0.001, *r* = 0.659), and GDF15 and hepcidin (*P* < 0.001, *r* = 0.453), as demonstrated in Figure 1.

## 4. Discussion

Iron homeostasis and erythropoietic activities appear to differ between thalassemia types and ethnic groups [23]. The globin chain imbalance effect resulted in chronic hemolytic and then ineffective erythropoiesis (IE), which correlated with the compensatory mechanism for anemia and hypoxia [1, 6]. In this study, most of the patients with newly diagnosed *β*-thalassemia major were well-nourished babies. This indicated that their anemia and hypoxia state did not affect their nutritional status. High SF and TS levels in newly diagnosed *β*-thalassemia major indicated that even before transfusions, the patients had a high level of iron in the body system. The definite mechanisms remain unclear, but IE might be the answer, as a high GDF15 level is observed. Practically, this finding warned time to start delivering iron chelation therapy not only considered by the number of blood transfusions. The ideal management approach is to avoid iron buildup from the outset because once patients are iron overloaded, decreasing iron to safe levels may take several years of consistent chelation treatment [26].

Erythropoiesis plays a role in the regulation of hepcidin, and increased erythropoietic activity can suppress hepcidin synthesis, which can lead to the release of plasma iron from iron storage in the liver and macrophages. Previous experimental studies in humans showed that stimulated erythropoiesis directly induced hepcidin suppression [27, 28]. It was reported that hepcidin production is paradoxically suppressed in *β*-thalassemia major during active erythropoiesis, while elevated serum iron levels normally stimulate hepcidin synthesis [11, 21].

In *β*-thalassemia major, the hepcidin level seems to change depending on the mechanism. When anemia and erythropoietic activities predominate, it results in hepcidin deficiency, but in the presence of iron overload, hepcidin expression can increase. However, a previous study showed that the driving force for erythropoiesis was apparently more dominant in influencing hepcidin levels when iron overload and anemia coexisted [3, 18, 20]. It was suggested that IE in *β*-thalassemia inhibits hepcidin synthesis by the release of GDF15, as described in Table 2. A high level of GDF15 indicates increased erythropoietic activity in the bone marrow. This condition was also reported in congenital dyserythropoietic anemia [22, 29]. Another previous study also showed that GDF15 was isolated during the final stages of erythroid differentiation, which suggests that GDF15 is secreted by erythroid precursors undergoing cell death. These findings confirmed that GDF15 was not produced by proliferating erythroid precursors but rather by apoptotic erythroid cells, as in *β*-thalassemia [10]. Therefore, GDF15 may limit hepcidin synthesis when erythroid precursors undergo cell death [6]. Furthermore, the GDF15 level may be an excellent in vivo biomarker of IE in *β*-thalassemia major.

Blood transfusion is considered to be one of the causes of iron overload in *β*-thalassemia major. In patients with chronic transfusion, it is expected that the hepcidin level is significantly higher than in patients with nonchronic transfusion due to an increased iron load in addition to high erythropoietic activity [3]. Gardenghi et al. reported that organ iron content in mice with thalassemia increased over time with increasing age [30]. Huang et al. stated that hepcidin levels tend to be low in multitransfused *β*-thalassemia. They also concluded that patients with severe *β*-thalassemia had a lower hepcidin level than healthy controls. Similarly, the hepcidin level was correlated with IE but not with the iron load [23]. El Beshlawy also stated that hepcidin levels in all types of congenital chronic hemolytic anemia, including *β*-thalassemia major, were lower than those in controls [3]. In contrast, the higher hepcidin level in *β*-thalassemia was marked in this study and in other reported studies. Haghpanah et al. stated that the regulation of hepcidin in *β*-thalassemia patients was more influenced by erythropoietic activity than iron load. Serum hepcidin levels were higher in *β*-thalassemia major patients than in *β*-thalassemia intermedia patients and controls [31]. Kaddah et al. reported that *β*-thalassemia major and *β*-thalassemia intermedia patients have high serum hepcidin and concluded that information on hepcidin levels in patients with *β*-thalassemia may help in identifying the most severely affected patients and predicting and monitoring parenchymal iron overload [20].

The hepcidin level was decreased in the interval between transfusions because it had no transfusion effect. The accumulation of iron in the absence of blood transfusions may be due to inadequate suppression of hepcidin by the erythropoietic mechanism [10, 21]. In this condition, the reduction in hepcidin led to increased absorption of iron and significantly contributed to iron overload in patients [28]. Apparently, there was an overlap of hepcidin regulation between IE-hypoxia and iron absorption. Most cases of iron overload demonstrate iron dysregulation and erythroid signaling, resulting in insufficient expression of hepcidin to maintain normal iron homeostasis. This condition may be different in newly diagnosed and chronic severe *β*-thalassemia [6, 18].

A positive correlation was found between SF-TS, SF-hepcidin, TS-hepcidin, SF-GDF15, and TS-GDF15 in newly diagnosed *β*-thalassemia major patients (*P* < 0.001). This is in line with Huang et al., who reported a positive correlation between SF and hepcidin in TDT patients [23]. However, in this study, hepcidin also had a positive correlation with GDF15, which was different from the results of Huang et al. This might be a characteristic of newly severe *β*-thalassemia patients who were different from chronic transfused severe *β*-thalassemia patients. A previous study in patients with multitransfused *β*-thalassemia did not find any significant correlation between serum hepcidin and SF, total iron-binding capacity (TIBC), and Hb. The mechanism remains unclear, but body compensation might be responsible [22].

Iron homeostasis and erythropoietic activity appeared to be different between newly diagnosed *β*-thalassemia major and chronic *β*-thalassemia patients who had received multiple transfusions. This study provided evidence that *β*-thalassemia major showed a higher level of IE based on an increased level of GDF15 and tended to have increased iron load based on the rise in SF and TS levels. However, it was estimated that in newly diagnosed *β*-thalassemia, major hepcidin suppression had not occurred, as evidenced by the level of hepcidin still being normal and even higher than in nonthalassemic children. A high level of iron body status might be due to other mechanisms not related to hepcidin suppression. In newly diagnosed *β*-thalassemia major, massive IE, which causes an increase in iron absorption, causes the liver to produce hepcidin in response to iron load. Otherwise, in chronic *β*-thalassemia, abundant hepatic storage of iron was a risk factor for liver disorders, even liver dysfunction, which may likely contribute to the decrease in hepcidin production.

In addition, there are many factors that influence the complex interactions of iron homeostasis in *β*-thalassemia major, including genetic background and race [23, 28].

### 4.1. Limitations of This Study

In this study, due to cost constraints, not all parameters related to iron homeostasis, such as EPO, soluble transferrin receptor (sTfr), erythroferrone (ERFE), and LIC, were measured. The subject's blood samples were only taken once, and therefore, there were some factors affecting iron metabolism and homeostasis that could be missed. All subjects were of the Sundanese ethnic group and apparently did not represent the entire Indonesian population in which *β*-globin mutation types in severe *β*-thalassemia were different between each region.

## 5. Conclusions

In newly diagnosed *β*-thalassemia major, ineffective erythropoiesis was characterized by an increase in GDF15 levels and an increase in iron levels due to a high rate of iron absorption. However, iron homeostasis appears to be physiologically indicated by the level of hepcidin, which is higher than that in healthy children. This finding is quite different from those in chronic *β*-thalassemia major, which causes a high level of erythropoietic activity with a high level of GDF15, resulting in hepcidin suppression. Further studies are needed to elaborate on this phenomenon.

## Figures and Tables

**Table 1:** Characteristics and clinical parameters of subjects with newly diagnosed severe *β*-thalassemia and controls.

	Case group (*N* = 41) *β*-Thalassemia major	Control group (*N* = 50)	*P*
Sex			0.322
Male	24	24	
Female	17	26	
Age (months old)			0.658
0–12	33	37	
12–24	8	13	
Median	7.0	8.5	
Range	2–24	2–28	
Nutritional status			0.112
Normal	30	35	
Mild malnutrition	10	15	
Moderate malnutrition	1	0	
Hemoglobin (g/dL)			<0.001
Median	5.0	11.7	
Range	1.6–7.8	10.6–13.5	
Serum ferritin (ng/mL)			<0.001
Median	315.0	29.0	
Range	83.6–1071.0	12.0–186	
Transferrin saturation (%)			<0.001
Median	70.6	16.5	
Range	28.5–98.7	8.8– 43.0	
Hepcidin serum (ng/mL)			<0.001
Median	251.0	123.1	
Range	114.5–363.5	5.6–349.5	
GDF15 serum (pg/mL)			<0.001
Median	2,095.3	342.4	
Range	149.3–9,586.3	350.08–436.99	

GDF15 = growth differentiation factor 15.

**Table 2:** A comparison of iron regulation in normal, untranfused, and transfused *β*-thalassemia major.

Condition	Erythropoiesis	GDF15	Hepcidin	Iron absorption
Normal	Normal	Normal	Increases when the iron body level increases or with inflammation	Decreases
			Decreases with iron deficiency or hypoxia	Increases
Untranfused *β*-thalassemia major	Increased IE	Increases	Decreases	Increases
Transfused *β*-thalassemia major (regular transfusion)	Decreased IE	Decreases	Increases	Decreases

GDF15 = growth differentiation factor 15; IE = ineffective erythropoiesis.

## Data Availability

The data used to support the findings of this study are available from the corresponding author upon request.
